# Surgical Repair of Complex Aortopulmonary Window: A Case Study

**DOI:** 10.21470/1678-9741-2017-0231

**Published:** 2018

**Authors:** Jigang He, Dan Yan, Beibei Li, Hongrong Li

**Affiliations:** 1 Cardiovascular Surgery, The First People's Hospital of Yunnan Province, Kunming, China.

**Keywords:** Aortopulmonary Septal Defect, Heart Defects, Congenital, Surgery, Cardiovascular Surgical Procedures

## Abstract

Aortopulmonary septal defect, also known as the aortopulmonary window, is a rare congenital macrovascular malformation. This case involves a 9-year-old boy with aortopulmonary septal defect (type I combined with type IV). Before surgery, milrinone and alprostadil were used to counteract high lung pressure. Surgery was performed under cardiopulmonary bypass, following which the pulmonary pressure decreased. The aorta was cut, and the right pulmonary artery opening was connected with the main pulmonary artery septal defect using polyester patch. An internal tunnel was made, and the deformity correction was completed. The child exhibited normal postoperative recovery with no discomfort. A complex aortopulmonary window is a rare condition that can be treated successfully with appropriate preoperative and surgical management.

**Table:** 

Abbreviations, acronyms & symbols	
CT	= Computed tomography
ECHO	= Echocardiography
EKG	= Electrocardiography
HTK	= Histidine-tryptophan-ketogluterate
PCO_2_	= Partial pressure of carbon dioxide
PO_2_	= Partial pressure of oxygen

## INTRODUCTION

Aortopulmonary septal defect, also known as the aortopulmonary window, is a rare congenital macrovascular malformation. According to Stansel et al.^[1]^, only less than 100 cases have been reported to date. The defect is located between the ascending aorta and the common pulmonary artery and is associated with a rapid progression of pulmonary arterial hypertension, unless it is corrected surgically^[2]^. Its pathophysiology and clinical manifestations resemble those of patent ductus arteriosus. The aortopulmonary window is used for surgery and interventional blockade treatment. Reports indicate that even in patients with severe pulmonary arterial hypertension with elevated pulmonary vascular resistance, aortic pulmonary window can be successfully corrected and most patients had satisfactory long-term outcomes^[3]^. Moreover, it is also safe in children beyond infancy (age range 14 months-12 years) with acceptable early and mid-term outcomes^[4]^. We report a case where surgical repair of complex aortopulmonary window was successfully performed in a nine-year old boy.

## CASE REPORT

**Fig. 1:**
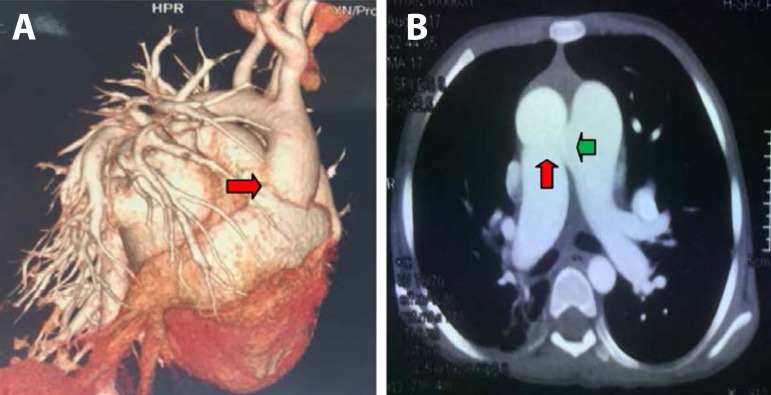
3D reconstruction and cross-sectional map of the heart. A) Red arrow points to the right pulmonary artery originating from the ascending aorta. B) Red arrow points to the right pulmonary artery originating from the ascending aorta. Green arrow points to the aortopulmonary septal defect.

A nine-year-old male child, with a prior history of heart murmur at birth, was admitted to our hospital on July 2, 2017. He presented with minor symptoms, comprising low activity, frequent cold compared to normal children, minor cyanosis and tachypnea after exercise. At the time of presentation, the heart rate was 105 beats/min, while the respiratory rate and blood pressure were 20 breaths/min and 109/67 mmHg, respectively. Short systolic II/6 rough noises were heard at the left margin of 4-5 ribs of the sternum, along with signs of loud P2 pulmonary hypertension. Post-admission arterial blood gases were 47.3 mmHg (partial pressure of oxygen = PO_2_) and 29.2 mmHg (partial pressure of carbon dioxide = PCO_2_). The computed tomography (CT) scan of the large thoracic and abdominal vessels showed the following findings: no aortic coarctation occurred; the right pulmonary artery originated from the ascending aorta; and the defect, which measured approximately 2.10-2.16 cm, was located between the pulmonary and the main artery (Figures 1A and 1B). Echocardiography (ECHO) result showed an aortopulmonary window (type I). The abnormal pathway (width: 1.73-2.09 cm) was found between the ascending aorta and the pulmonary artery. The estimated pulmonary artery pressure was 71 mmHg, with shunting from left to right. The electrocardiography (EKG) result also indicated that a high-voltage sinus rhythm occurred at the left ventricle and that the T wave changed on the anterior wall. Preoperative pulmonary artery resistance was of 7 Wood units, which was measured during the heart catheterization exam. Alprostadil (10 µg, with 0.17 µg/min intravenous infusion) and milrinone (0.375 µg/kg.min, with 24 h continuous intravenous infusion) were administered to reduce the pulmonary arterial pressure. Blood gases were found to be 55.7 mmHg (PO_2_) and 29.6 mmHg (PCO_2_) when retested after 14 days of hospital admission. Meanwhile, EKG results again showed the aortopulmonary window (type I) with an abnormal pathway (width: 1.73-2.09 cm) between the ascending aorta and the pulmonary artery. The estimated pulmonary artery pressure was 63 mmHg, with shunting from left to right. The precordial murmur was louder than that before admission.

**Fig. 2:**
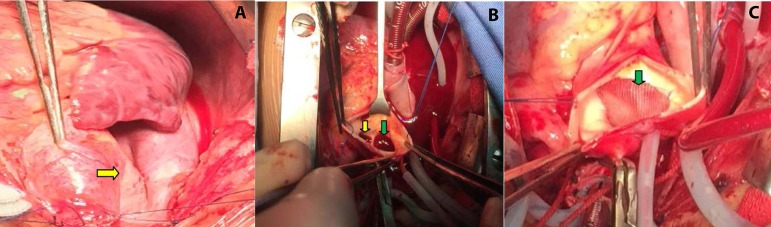
Surgical images showing polyester patch was used to separate the right pulmonary artery from communicating to the aorta and to correct the aortopulmonary septal defect. A) Yellow arrow points to the right pulmonary artery originating from the ascending aorta. B) Green arrow points to the right pulmonary artery opening; yellow arrow points to the defect between the ascending aorta and the pulmonary artery. C) Patch separates the right pulmonary artery opening from the aorta pulmonary through the defect.

**Fig. 3:**
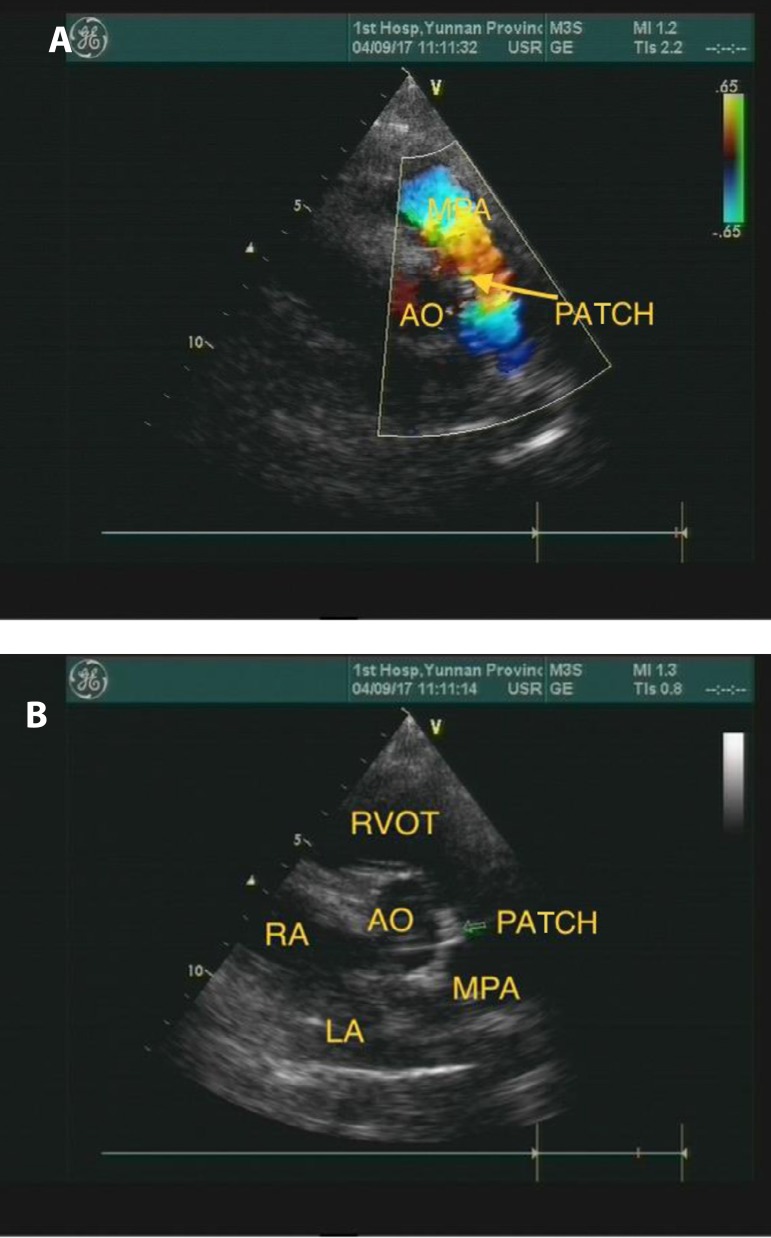
Post-operative ECHO images. AO=aorta; LA=left atrium; MPA=main pulmonary artery; RA=right atrium; ROVT=right ventricular outflow tract

The patient underwent aortopulmonary septal defect repair under general anesthesia 15 days after admission. Aortic cannulation was placed below the right arm artery (cannulation of the superior and the inferior vena cavae). The ascending aorta was blocked at 35ºC degrees, longitudinally cut, and cardiac protective solution [custodiol / histidine-tryptophan-ketogluterate (HTK)] was poured under direct vision. The review indicated that the right pulmonary artery originated from the ascending aorta. The defect, which measured approximately 2.0-2.5 cm, was observed between the ascending aorta and the pulmonary artery. The opening of the right pulmonary artery was connected to the defect. We used a polyester patch to separate the right pulmonary artery from communicating to the aorta and to correct the aortopulmonary septal defect (Figures 2A, B and C). The aortic incision was then sutured and the heart re-warmed to 37ºC. The ascending aorta was opened after full exhaustion, and the heart was automatically resuscitated. The use of alprostadil and milrinone was continued to reduce lung pressure. The tracheal intubation was removed 4 hours after anesthetic awareness. Postoperative recovery was successful. The heart color ultrasound on the 10^th^ day presented the following result: no residual shunt was observed after the repair of aortopulmonary septal defect, the pressure in the pulmonary artery was slightly elevated, and the estimated pulmonary artery pressure was 42 mmHg (Figure 3). After the surgery, the patient refused re-catheterization and ultrasound was performed for measuring pulmonary artery resistance. The patient was discharged from the hospital with indication to take oral captopril. The patient exhibited no symptom of discomfort during the follow-up visit.

## DISCUSSION

The aortopulmonary window is an extremely rare cardiac anomaly resulting from incomplete development of conotrucal septum^[5-7]^, with an incidence rate of 0.2-0.6% of all congenital heart diseases^[4]^. It is categorized into three types by Mori: (1) type I or proximal defect, which is located 1-1.5 cm away from the aortic valve, (2) type II or distal defect, which appears and disappears between the distal ascending aorta and the pulmonary artery, and (3) type III or the complete defect^[8]^. At present, the right pulmonary artery that originates from the aorta is classified as type IV defect^[9,10]^. In this case study, the patient suffers from an extremely rare deformity, *i.e*., type I combined with type IV. The survival of patients with aortopulmonary window depends on the defect size and the pulmonary vascular resistance. The repair of aortopulmonary window is ideally performed in infancy, before irreversible pulmonary arterial hypertension has developed^[4,11]^. In cases where severe defects remain untreated, most patients may die of heart failure and only a few may live until adolescence or adulthood. Undergoing surgery (in most cases) or vascular occlusion (in small restrictive defects) to achieve early closure is the best treatment to guarantee the survival of patients^[12]^. In this specific case, the defect went unnoticed until 9 years of age, and he could have survived until late childhood without active treatment. The major decision factors in the treatment of children presenting with defects beyond infancy are the assessment of operability, the postoperative management of pulmonary hypertension, and long-term outcomes^[4]^. Previous reports demonstrated favorable early and long-term outcomes after surgical correction, regardless of age or pulmonary vascular resistance^[2,3]^. Based on these reports, we decided to proceed with the surgical repair of the defect in the subject. Reduction in the pre-operative pulmonary artery pressure was achieved by the administration of milrinone and alprostadil. The surgery was uneventful and the post-operative recovery successful. This experience demonstrates that we can reduce pulmonary artery pressure by actively reducing the lung pressure and underscores the importance of surgical repair even in complex defects. As suggested previously, our report strongly supports that surgical repair should be offered as soon as the diagnosis is established, regardless of the patient's age. In addition, for patients who are responsive to pulmonary hypertension treatments *i.e*. with reduced blood pressure in the lung, surgery should be considered. Patients with an aortopulmonary window should immediately undergo surgery, and the surgical method should be determined based on the type of preoperative deformity.

## CONCLUSION

A complex aortopulmonary window is a rare condition that can be treated successfully with appropriate preoperative and surgical management.

**Table:** 

Authors' roles & responsibilities	
JH	Carried out operation and drafted the manuscript; participated in the design of the study and performed data collection; conceived of the study, and participated in its design and coordination and helped to draft the manuscript; final approval of the version to be published
DY	Participated in the design of the study and performed data collection; final approval of the version to be published
BL	Participated in the design of the study and performed data collection; final approval of the version to be published
HL	Conceived of the study, and participated in its design and coordination and helped to draft the manuscript; final approval of the version to be published
